# Industrialization of sandwich composite panels for portable outdoor tabletops

**DOI:** 10.1371/journal.pone.0353571

**Published:** 2026-07-09

**Authors:** Wen Yu, Robert Langer

**Affiliations:** 1 Topsun National Industrial Design Center, Zhejiang Hengfeng Top leisure Co., Ltd., Hangzhou, Zhejiang Province, P. R. China; 2 Faculty of Natural Sciences II, Institute of Chemistry, Martin Luther University Halle-Wittenberg, Halle, Germany; King Mongkut’s University of Technology North Bangkok, THAILAND

## Abstract

Sandwich composite panels offer a promising pathway for developing lightweight yet mechanically robust portable outdoor tabletops, but their industrialization outside traditional aerospace and transportation sectors remains challenging. This study investigates how sandwich composites can be adapted for mass‑market outdoor tabletops by linking market‑driven requirements to engineering design decisions. Five representative panel configurations were developed, varying in core materials, face‑sheet systems, surface finishing, edge‑sealing strategies, and fastening solutions. A structured evaluation matrix was established to assess both material‑level and product‑level performance, aligned with customer‑relevant requirements, applicable standards, and company‑defined acceptance criteria. Results show that industrial feasibility is governed not by any single metric but by the coupled interactions among material selection, surface durability, moisture‑resistant edge sealing, fastening reliability, and manufacturability-cost trade‑offs. Blind rivets emerged as a practical fastening solution, while surface durability and thermal stability were strongly influenced by the combined behavior of finishing chemistry and substrate. The findings establish practical architecture-performance relationships and outline viable industrialization pathways for mass‑market portable outdoor tabletops based on sandwich composite panels.

## 1. Introduction

Sandwich composite structures have long been refined in high‑performance sectors where weight reduction is a primary design driver. Originally developed for aerospace and other transportation applications, sandwich panels combine thin, high‑strength facings with a lightweight core to achieve exceptionally high stiffness‑to‑weight ratios [1–4]. In aerospace, for example, honeycomb‑ or foam‑cored sandwich skins are widely used in wing and fuselage structures [5,6]. Similar principles have been adopted in rail and marine applications, where reduced structural mass directly improves operational efficiency [7–9]. Decades of research have advanced manufacturing processes (e.g., autoclave curing, co‑curing) and material systems (e.g., carbon fibers, polymer foams, metal or polymer honeycombs), lowering costs and expanding design knowledge [10–12]. As a result, sandwich composites have reached a point where their cost-performance ratio enables broader civilian use. Recent reviews indicate growing interest in applying sandwich structures to civil infrastructure, driven by the need for lightweight and durable materials [13–15].

A major emerging opportunity lies in leisure and portable outdoor furnishings, particularly tabletops. The global outdoor furniture market reached USD 56 billion in 2025 and is projected to exceed USD 92.08 billion by 2034 [16], while the portable outdoor furniture segment is expected to grow from USD 6.3 billion in 2023 to USD 11.5 billion by 2032 [17]. These markets are shaped by trends such as urban outdoor living, al fresco workspaces, and recreational events, all of which demand lightweight, durable, and weather‑resistant products. Outdoor tables must withstand rain, sunlight, temperature swings, and mechanical wear while remaining easy to move and assemble [16,17]. Industry reports emphasize that consumers expect multifunctional outdoor products capable of enduring harsh weather with minimal maintenance [16]. Manufacturers are responding with designs that are lightweight yet robust, with many outdoor‑gear companies focusing on “crafting lightweight, environment‑friendly, durable fixtures” for this growing market [16]. These requirements, which are lightweight, high strength, weather resistance, and adaptability, align closely with the inherent advantages of sandwich composites. Recent work shows that hybrid natural/glass fiber systems can offer a favorable trade off between lightweighting and impact absorption for consumer grade components [17].

**Fig 1:**
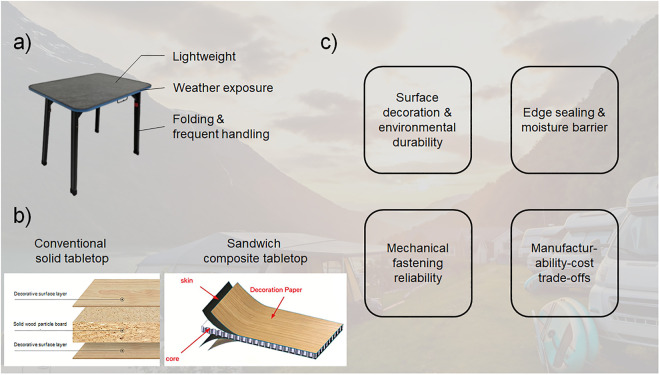
Conceptual illustration of sandwich composite panels for portable outdoor tabletops. (a) Typical usage scenarios highlighting requirements for low weight, weather resistance, and frequent folding. (b) Cross‑sectional comparison between a conventional solid tabletop and a sandwich composite tabletop, showing face sheets, lightweight core, edge regions, and fastening zones. (c) Mapping of application‑driven requirements to the primary engineering challenges addressed in this study, including surface durability, edge sealing, fastening reliability, and cost-manufacturability considerations.

However, unlike aerospace applications, the outdoor consumer market imposes strict cost and usability constraints and competes directly with low‑cost woods and plastics. Market data shows that wood remains the dominant outdoor furniture material due to its durability, aesthetics, and cost‑effectiveness [18]. To succeed in this market, sandwich composites must match or exceed wood’s value proposition. Rather than repurposing aerospace‑grade designs, new research is required to address the distinct challenges of adapting sandwich composites to outdoor tabletops. Consumer‑grade furniture must combine visual appeal with environmental resilience, ensure reliable edge protection, provide secure fastening for collapsible frames, and meet cost targets suitable for mass‑market production. Fig 1 summarizes the application context, structural characteristics, and key engineering challenges associated with adapting sandwich composite panels for portable outdoor tabletops.

### 1.1. Surface decoration and environmental durability

Where aerospace and rail panels typically use bare‑metal or plain laminated finishes, consumer tabletops require refined surface aesthetics, such as wood‑grain textures, matte or semi‑gloss coatings, and full‑color graphics, combined with resistance to ultraviolet (UV) radiation, moisture, abrasion, and staining. Decorative layers must maintain adhesion under temperature cycling (−20 °C to +60 °C) and wet-dry fluctuations without yellowing, chalking, or peeling [19]. Similar durability-lightweighting trade-offs have been investigated in outdoor hybrid composites, where combined UV and moisture exposure progressively degrades both mechanical and viscoelastic properties [20]. Developing or qualifying surface treatments that balance long‑term weatherability with consumer‑grade aesthetics is therefore a foundational challenge.

### 1.2. Edge banding and moisture barrier

Exposed sandwich‑panel edges are inherently vulnerable to moisture ingress, which can lead to core saturation, adhesive degradation, and premature delamination. Outdoor tabletops require edge treatments that form a continuous, impermeable barrier while preserving the panel’s visual lines and low weight. Candidate solutions, including flat edge banding and wrapped metal profiles, must be evaluated for long‑term moisture resistance, UV stability, and installation efficiency in mass production.

### 1.3. Mechanical fastening

Sandwich‑panel cores based on metal or polymer honeycombs and closed‑cell foams provide limited substrate for conventional screws or bolts. Reliable leg‑mount points therefore require engineered fastening strategies, such as embedded threaded inserts, bonded reinforcement blocks, blind‑rivet anchors, or hybrid fixtures. Previous studies on blind rivets in sandwich structures have focused primarily on single‑rivet pull‑out behavior in polyisocyanurate (PIR) foam panels with steel or laminate facings, highlighting the influence of facing stiffness and core adhesion [21]. In consumer outdoor products, however, fastening systems must provide adequate pull‑out and shear strength under static load, repeated folding cycles, and accidental impacts, while enabling straightforward field assembly without specialized tools.

### 1.4. Cost-performance trade-offs in material selection

Although aerospace‑grade metals and advanced polymer cores offer superior performance, their material and processing costs often exceed consumer price thresholds [22–24]. Outdoor tabletops must balance performance benefits (e.g., lightweighting, durability) with production economics. Material candidates, such as aluminum versus polymer cores, metal versus polymer or natural‑veneer skins, and standard versus specialty adhesives, must be assessed not only for mechanical and environmental performance but also for raw‑material cost, processing complexity, and supply‑chain maturity.

This study investigates the potential of sandwich‑structured tabletop panels by developing five prototype configurations spanning a range of core materials, face‑sheet types, edge‑sealing strategies, and fastening solutions. These prototypes underwent targeted performance evaluations, including surface durability, edge integrity, fastening fatigue, flexural strength, and environmental cycling, to identify architecture-performance relationships relevant to consumer‑ready outdoor panels. The broader context for this work is the ongoing transition from automated, efficiency driven composite manufacturing toward more agile, customer responsive production paradigms, as outlined in recent reviews of Industry 4.0 to 5.0 evolution in composite products [25]. The present study contributes to this transition by providing a structured, data anchored methodology for adapting sandwich composite technology to the specific cost, durability, and manufacturability requirements of the outdoor consumer market. The findings aim to provide design guidance for integrating advanced sandwich composites into portable outdoor tabletops, combining high‑performance materials with the practical requirements of everyday use.

## 2. Prototype panel designs & evaluation setup

This section summarizes the five prototype panel types used to investigate industrialization challenges, provides a compact configuration table, outlines the non‑proprietary fabrication principles, and describes the test program. The experimental program was designed to link architectural choices, for example, core family, face‑sheet family, edge treatment, and fastening approach, to measurable outcomes relevant to portable outdoor tabletops.

### 2.1. Prototype overview

Five representative panel configurations (Panels A-E) were selected to span a practical design space ranging from industry‑typical, low‑cost constructions to premium honeycomb solutions with varied surface options. Section 2.2 summarizes the five prototype configurations. A more detailed material specification table, including density and thickness ranges, is provided in Supporting Information (Table A in S1 File). These ranges are reported to provide quantitative context for the mechanical and durability comparisons that follow while protecting commercially sensitive information.

Panels A and B apply the sandwich principle to reduce mass relative to conventional solid‑wood or particle/plywood panels. These two configurations explore how sandwich concepts can deliver weight savings while maintaining familiar consumer surfaces (e.g., plywood or bamboo veneer) that are visually appealing and consistent with broader trends in wood‑based sandwich panel design [26,27].

Panels C and D both incorporate aluminum honeycomb cores and were designed to evaluate higher specific‑stiffness architectures. Their primary distinction lies in face‑sheet selection and finishing systems, enabling assessment of how different decorative or functional surfaces behave when paired with metal‑honeycomb cores. Both panels were produced using the same class of processing equipment.

Panel E represents a low‑cost exploration: a polymeric honeycomb core combined with a fiber‑reinforced polymer (FRP) skin and a thin decorative overlay. This configuration can be manufactured using lower‑cost, high‑throughput processes such as hot lamination. Panel E evaluates whether acceptable performance can be achieved at substantially reduced material and processing cost.

Collectively, Panels A and B represent sandwiching as a route to lighten traditional panels; Panels C and D probe premium honeycomb architectures and varied surface cosmetics; and Panel E examines low‑cost, high‑throughput options. This spread enables evaluation of how core selection, face‑sheet choice, edge treatment, and fastening strategy interact to determine bonding behavior, weather resistance, and fastening performance.

### 2.2. Summary table of configurations

**Table:** 

ID	Core Family	Face Family	Edge Treatment	Finish	Fastening Approach
**A**	Closed‑cell PET foam	Exterior plywood	Adhesive seal	Melamine‑impregnated paper	Direct self‑tapping screws
**B**	Aluminum honeycomb	Bamboo veneer	Bamboo veneer	Clear lacquer	Embedded wood/block reinforcement (screw)
**C**	Aluminum honeycomb	HPL laminate	Wrapped metal profile	Melamine‑impregnated paper	Plastic embedded inserts (screws)
**D**	Aluminum honeycomb	Metal sheet	Adhesive seal	UV‑cured print on metal	Blind rivets / plastic embedded inserts
**E**	Polymeric honeycomb	FRP + HPL overlay	Adhesive seal or wrapped metal profile	Melamine‑impregnated paper	Blind rivets / plastic embedded inserts

Note: exact thicknesses, adhesive chemistries, press temperatures, and supplier identities are proprietary and omitted. A general fabrication overview is provided below; full process parameters are available under confidentiality agreement.

### 2.3 Manufacturing overview and IP statement

To avoid disclosure of proprietary manufacturing know‑how, only general process descriptions are provided. No commercially sensitive information, such as machine specifications, detailed process parameters, adhesive formulations, or curing profiles, is included. All prototype panels were produced under a joint development agreement. Any proprietary process innovations generated during this project remain fully or jointly owned, and no confidential or sensitive parameters are disclosed.

Panel A. Decorative melamine‑impregnated paper was laminated to plywood using a standard hot‑press process, followed by cold‑press bonding to a polyethylene terephthalate (PET) foam core. Edge sealing was achieved using a conventional polyurethane reactive adhesive (PUR) flat‑edge‑banding system.

Panel B. The aluminum‑honeycomb core, screw‑holding inserts, peripheral bamboo frame, and top/bottom bamboo veneers were bonded using a conventional cold‑press process.

Panels C and D. Panels C and D share a similar lamination architecture. All layers were bonded using a cold‑press process. After curing, holes were drilled on the backside, adhesive was dispensed, and raised‑boss plastic inserts were affixed.

Panel C: The surface panel consists of melamine‑impregnated paper laminated onto high‑pressure laminate (HPL); edges were finished using aluminum profiles.

Panel D: The surface consists of an aluminum sheet bonded to the core, followed by UV ink printing; edges were sealed using a PUR flat‑edge‑banding system specifically designed for aluminum‑honeycomb panels.

Panel E. Melamine paper was first laminated to thin HPL, then hot‑pressed with a fiberglass sheet, and subsequently bonded to a polymer honeycomb core. Panel E can be finished with aluminum profiles (as in Panel C) or with shaped‑edge‑banding equipment for flat edges.

### 2.4 Evaluation and testing methods

The evaluation matrix included both material‑level and product‑level tests. Material‑level assessments examined surface durability, edge‑banding performance, fastening strategies, and substrate behavior. Product‑level assessments evaluated assembled table prototypes, focusing on joint shear fatigue and overall structural robustness. Standardized methods primarily followed GB/T 17657‑2022 for material‑level testing and EN 581‑3:2017 for product‑level testing. Where no applicable standard existed, internal methods were developed by adapting principles from established test standards; the specific adaptations and their rationale are described in the relevant subsections below. All internal methods underwent repeatability verification across multiple batches; the resulting coefficients of variation are reported alongside the results in Section 3.

#### 2.4.1 Surface-related tests.

Surface durability was evaluated using multiple GB/T 17657‑2022 methods and AATCC 186‑2015 for UV aging. For all rating‑based tests, higher grades indicate better performance.

1. **Resistance to abrasion (GB/T 17657‑2022 4.46):**
  Number of revolutions until first visible wear; higher counts indicate stronger abrasion resistance.
2. **Resistance to scratching (GB/T 17657‑2022 4.42):**
  Rated from Grade 1 (poor) to Grade 5 (no visible trace).
3. **Resistance to crazing (GB/T 17657‑2022 4.39):**
  Evaluated visually; higher grades represent better resistance.
4. **Resistance to dry heat (GB/T 17657‑2022 4.49):**
  Highest temperature (120–200 °C) at which the surface maintains Grade 5.
5. **Resistance to wet heat (100 °C) (GB/T 17657‑2022 4.50):**
  Rated 1–5; higher grades indicate improved humidity resistance.
6. **Resistance to staining (GB/T 17657‑2022 4.43):**
  Five standard contaminants applied and assessed; Grade 5 indicates complete resistance.
7. **Cross‑cut test (GB/T 17657‑2022 4.57):**
  Classified using national adhesion grid rating.
8. **Resistance to UV light (AATCC 186‑2015):**

Evaluated by visual color change and surface condition.

#### 2.4.2 Edge-banding tests.

**Fig 2:**
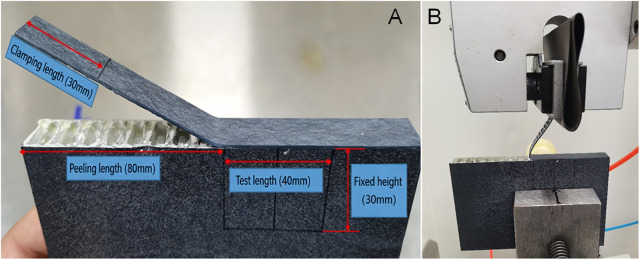
Internal peel‑test setup for evaluating edge‑band adhesion. (A) Specimen schematic showing test regions and dimensions. The specimen (150 × 100 mm) contains one sealed edge (150 mm). (B) Specimen mounted in a CRE tensile tester: the upper moving grip clamps the pre‑peeled section while the lower fixture secures the base during testing. A 80 mm pre‑peeled section was introduced manually; the upper 30 mm was clamped in the moving grip while the remainder was fixed. Tests were conducted on a CRE tensile tester at a constant crosshead speed of 50 mm/min. Average and maximum peel forces were recorded over the central peeling zone. Environmental pre‑conditioning (hygrothermal aging and water immersion) was applied prior to repeat testing.

A peel test protocol was developed for edge-band adhesion because no national or international standard specifically addresses edge banding on sandwich panels with cellular cores. The test methodology was adapted from the general principles of 90° peel testing for adhesives, as described in ASTM D6862 and ISO 11339:2022. Tests were conducted using a constant-rate-of-extension (CRE) tensile tester. The configuration is shown in Fig 2.

(1) Specimens: 150 × 100 mm with one 150 mm sealed edge
(2) Pre‑peeled length: 10 cm
(3) Test speed: 50 mm/min
(4) Metrics: average and maximum peel force over the central 3 cm peeling zone
(5) Pass criteria: Avg ≥ 30 N or Max ≥ 50 N

Environmental pre‑conditioning was applied to evaluate edge durability under simulated service conditions:

a) Hygrothermal aging: 72 h at 60 °C / 90% relative humidity (RH). This condition represents the upper bound of combined thermal and moisture stress on outdoor tabletops in summer climates and was selected to avoid exceeding the service temperature limits of the wood-based and thermoplastic constituents, consistent with the test conditions of accelerated aging described in ASTM D1151.
b) 24 h water immersion at 20 °C

#### 2.4.3 Fastening-related tests.

Fastening performance was assessed using both vertical (pull‑out) and horizontal (shear‑fatigue) loads. Representative failure‑mode images for pull‑out resistance tests and horizontal‑fatigue staircase tests are provided in the Supporting Information (S1 and S2 Fig).

1. **Axial withdrawal of screws (GB/T 17657‑2022 4.21):**

Applicable to panels allowing direct screw insertion (Panels A and B).

2. **Pull‑out resistance (internal method):**

**Fig 3:**
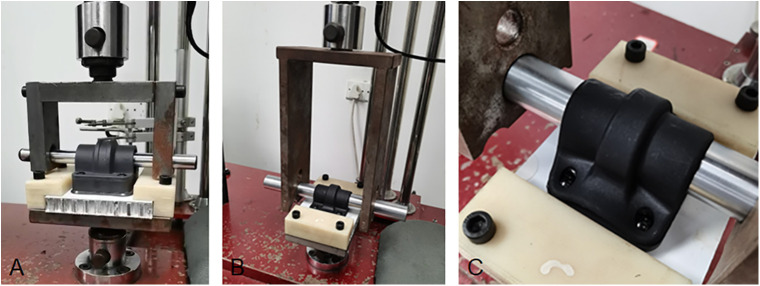
Pull‑out resistance test for embedded fastening inserts, shown from three complementary perspectives. Square specimens (100 × 100 mm) with a centrally embedded plastic insert were mounted on a dedicated fixture to ensure axial alignment. A vertical tensile load was applied at 15 mm/min until screw pull‑out or specimen failure occurred. The maximum recorded force was defined as the pull‑out strength.

Applied to screws, embedded plastic inserts, and blind rivets. The method was adapted from the screw withdrawal principles of GB/T 17657−2022 4.21, extended to accommodate non-screw fasteners and bonded inserts. The specimen geometry and loading setup are shown in Fig 3.

(1) Specimens: 100 × 100 mm with centered insert
(2) Loading rate: 15 mm/min
(3) Maximum force recorded as pull‑out strength

Representative failure modes observed during pull-out resistance testing are provided in Supporting Information (S1 Fig).

3. **Horizontal‑fatigue staircase test (product‑level):**

The staircase protocol was adapted from EN 581−3 test 5 to represent the repeated folding and opening loads experienced by outdoor tables. The protocol was applied in successive load stages:

a) 100 N / 5000 cycles
b) 150 N / 10,000 cycles
c) 200 N / 20,000 cycles
d) Additional steps until failure

Panels sustaining more stages or cycles exhibit greater shear‑fatigue durability.

#### 2.4.4 Substrate-related tests.

1. **Impact resistance with small ball (GB/T 17657‑2022 4.53):**

Drop height: 210 mm; performance ranked by visible change.

2. **Modulus of rupture (MOR) (GB/T 17657‑2022 4.7):**

Evaluated at room temperature and after hygrothermal conditioning (72 h at 60 °C / 90% RH), using the same accelerated aging condition applied in edge-band testing (Section 2.4.2).

3. **Water‑immersion test for bond durability (GB/T 17657‑2022 4.19):**

Three conditions of escalating severity were applied:

a) Condition c: 35 °C water / 2 h + 63 °C drying / 3 h
b) Condition b: 63 °C water / 3 h + 63 °C drying / 4 h
c) Condition a: boiling water / 4 h + 63 °C drying / 20 h + boiling water / 4 h + 63 °C drying / 3 h

Best case: “no change” after condition (a).

4. **Heat‑induced deformation (internal method):**

**Fig 4:**
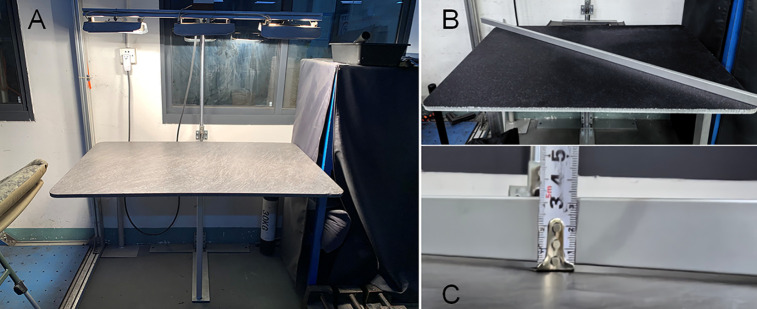
Heat‑induced deformation test used to simulate outdoor solar exposure. (A) Test setup showing three 1000‑W tungsten lamps arranged with 175‑mm spacing, and specimens placement at 600 mm from the heat source; the overall irradiation rig and specimen layout are shown. Each irradiation cycle consisted of 1 h exposure followed by a 10‑min cooling interval, repeated three times. (B) After irradiation, straight‑edge bars were placed along diagonal and orthogonal directions to measure out‑of‑plane deformation. (c) Deformation measurement close-up.

Developed to simulate asymmetric solar heating, which is the primary heat load on outdoor tabletops. The general testing methodology is consistent with the asymmetric thermal loading method used in ISO 4892 series artificial weathering standards, with adjustments made for tabletop-scale specimens. The irradiation setup is illustrated in Fig 4.

(1) Three 1000 W tungsten lamps
(2) Specimen placed 600 mm from lamps
(3) Three irradiation cycles (1 h exposure + 10 min pause)
(4) Deformation measured using straight‑edge bars and feeler gauges

Pass criteria: a) no cracking, blistering, or surface defects b) maximum deflection ≤ 0.5% of panel length

## 3 Results and discussion

This study aimed to develop and validate the key technologies required for industrializing sandwich composite panels for outdoor table applications. Using a structured evaluation matrix, we reported representative values obtained from repeated measurements and benchmarked them against technical requirements gathered from outdoor‑product clients, industry‑derived performance expectations, relevant national standards (GB/T series), and internally defined acceptance criteria. Performance was evaluated across four challenge dimensions:

(1) surface decoration and weatherability,
(2) edge sealing and moisture protection,
(3) mechanical fastening strategies, and
(4) material selection and cost-manufacturability trade‑offs.

The following sections analyze how the methods and test results inform engineering decision‑making for industrialization, identify challenges that can be effectively resolved, and highlight priority areas for continued development.

### 3.1 Surface decoration and weatherability

Surface durability is a critical requirement for outdoor tabletops because the decorative layer serves not only an aesthetic function but also acts as the primary barrier against ultraviolet radiation, moisture, temperature fluctuations, and household contaminants. Table 1 summarizes the performance of the five panel configurations across abrasion resistance, scratch resistance, crazing, UV aging, stain resistance, temperature and humidity exposure, and coating adhesion.

**Table 1:** Surface performance of the five sandwich panel configurations.

Test Item	A PET Foam + Plywood	B Al‑Honeycomb + Bamboo	C Al‑Honeycomb + HPL	D Al‑Honeycomb + Al plate	E Plastic Honeycomb + FRP + HPL
Resistance to abrasion (revolutions)	200	220	90	50	165
Resistance to scratching (grade)	1	1	1	1	1
Resistance to crazing (grade)	5	5	5	5	5
Resistance to dry heat (Highest temperature at which the surface maintains Grade 5) (°C)	120	120	160	200	160
Resistance to wet heat (100℃) (grade)	4	5	3	5	5
Resistance to staining (grade)(acetone/ coffee/ NaOH/ H₂O₂ / shoe polish)	5,5,5,5,4	5,5,5,5,4	5,5,5,5,5	5,5,5,5,5	5,5,4,4,4
Cross-cut test (grade)		6		6	
Resistance to UV light (grade)	4-5	5	4-5	4	4-5

Surface performance was evaluated using abrasion resistance, scratch resistance, crazing resistance, dry-heat resistance, wet-heat resistance, stain resistance, coating adhesion, and UV-aging resistance tests. Surface-property tests were conducted on one batch of three specimens per panel (n = 3), except UV-aging tests for Panels A and E, which were conducted on two batches of three specimens each (total n = 6). Results are reported according to the relevant grading systems or threshold temperatures defined in the referenced test methods.

Several surface-related properties showed little differentiation among the panel architectures. Coating adhesion applied only to finishing systems involving a paint layer, and both Panel B and Panel D demonstrated excellent adhesion performance. Scratch resistance and crazing resistance were identical across all panels (Grade 1 and Grade 5, respectively). Stain resistance was also generally high, with only minor reductions under selected reagents. These results indicate that all five surface systems satisfy the baseline durability requirements typically expected for outdoor consumer furniture. Consequently, the more meaningful distinctions arise from UV-aging, abrasion resistance, and thermal resistance behavior, where the influence of both surface chemistry and substrate architecture becomes evident.

UV-aging and Abrasion resistance performance were both governed primarily by the outermost functional layer. UV aging performance showed a clear dependence on the finishing system. Painted and UV‑protected surfaces (Panel B) performed best, followed by melamine‑impregnated decorative papers (Panels A, C, and E). UV‑inkjet systems (Panel D) showed the lowest stability under UV exposure. Abrasion resistance followed a similar trend: Panel B exhibited the highest resistance, melamine‑based surfaces showed intermediate performance, and Panel D showed the lowest resistance. Prior studies have shown that the abrasion resistance and lightfastness of melamine-impregnated papers depend on base-paper quality, printing ink composition, resin content, curing conditions, and the presence of ultraviolet absorbers or light stabilizers added during impregnation. Differences among suppliers in any of these factors can produce performance variation of this magnitude without implying a fundamental difference in panel architecture. Panel D’s low abrasion and UV resistance, despite its aluminum substrate, reflects the susceptibility of its UV-cured decorative print layer to progressive mechanical removal and photolytic degradation of the organic ink components.

Thermal resistance, in contrast, showed strong substrate dependence. Under dry heat, Panel D sustained Grade 5 at 200 °C, while Panels A and B were limited to 120 °C. This reflects the high thermal stability of aluminum, which does not soften, lose moisture, or undergo the localized thermal degradation that affects lignocellulosic materials. Panels C and E, both with non-wood substrates, reached 160 °C further supporting the role of the substrate in determining dry-heat tolerance. Under wet heat (100 °C), Panels B, D, and E achieved Grade 5, while Panel A dropped to Grade 4 and Panel C to Grade 3. Panel A’s moderate decline is attributable to its hygroscopic plywood faces, which absorb moisture and generate internal steam pressure at the face-core interface. Panel C’s sharp degradation to Grade 3 suggests a more severe mechanism. Panel C consists of a thin HPL skin directly bonded to an aluminum honeycomb core. Under 100 °C steam exposure, the high thermal conductivity of the aluminum core rapidly transfers heat to the bondline, creating a steep thermal gradient against the low-conductivity HPL face. If the cold-press adhesive possesses limited hydrothermal shear strength, or if the thin HPL skin contains residual stresses from manufacturing, the combination of thermal strain mismatch and localized vapor pressure at the interface can induce micro-cracking, blistering, and loss of surface integrity. Panel E avoids this failure mode by utilizing an FRP backing layer co-laminated with the HPL overlay over a polymeric honeycomb core. The FRP backing acts as a thermal and mechanical buffer: its lower thermal conductivity moderates the thermal gradient at the surface, and its hydrophobic, continuous structure blocks moisture accumulation at the HPL-substrate interface.

The combined dry-heat, wet-heat and UV-aging results demonstrate that surface durability should be viewed as a system-level property rather than a characteristic of the decorative layer alone. the same can produce different performance when paired with different substrates because thermal expansion, moisture uptake, stiffness, and dimensional stability influence the stresses transmitted to the surface. This interaction helps explain why outdoor furniture occasionally exhibits discoloration, blistering, or coating failure even when the decorative material itself performs well in isolated laboratory testing.

Collectively, these results show that no single finishing chemistry dominates across all dimensions. Instead, surface performance is a combined function of the decorative layer, the face sheet, and the core. For industrialization, this means that surface qualification cannot be reduced to a finish-only test: the substrate must be part of the evaluation. The single-batch screening data are fit for identifying non-compliant candidates, but deeper multi-batch characterization should be reserved for the final down-selected design.

### 3.2. Edge sealing and moisture protection

Edge sealing was evaluated only for Panels A, D, and E, which require post-applied flat edge bands. Panels B and C were excluded from the discussion for edge‑banding evaluation. Panel B does not require edge sealing due to its solid‑wood construction, and Panel C cannot be processed with flat edge‑banding because its thin HPL surfaces are too brittle for conventional PUR equipment. The discussion therefore focuses on flat edge‑banding systems, which have the highest market acceptance because they resemble indoor‑furniture aesthetics. Edge banding on sandwich structures is inherently challenging. Unlike aerospace sandwich panels, consumer outdoor tables require decorative, continuous, and touch‑safe edges.

Panel A delivered the highest peel strength and the narrowest scatter across all conditions. Its ambient mean exceeded 130 N with a coefficient of variation (CV) below 1%, and it retained over 90% of this strength after both aging regimes. This performance reflects the continuous, closed-cell bonding surface of the PET foam and the strong adhesion of PUR to the plywood substrate. In contrast, Panels D and E, both honeycomb structures, showed substantially lower peel strength and far wider scatter. Panel D exhibited a CV of approximately 29%, while Panel E reached roughly 38%, with mean values roughly half to one-third of Panel A’s.

The large CV of the honeycomb panels stems from a geometric limitation: only the thin cell walls provide bonding area, creating a discontinuous and irregular adhesive line. This alone explains the difference between Panel A and D, since they share the same PUR adhesive chemistry. Panel E’s further increase in variability is attributable to the chemical: PP-based hot-melt adhesives, while compatible with the PP core, generally exhibit lower cohesive strength and greater sensitivity to application temperature than PUR systems. The combination of a discontinuous bond line and a less robust adhesive produces both lower absolute strength and larger batch-to-batch and within-batch variability.

Accelerated aging amplified these differences. After water immersion, Panel A retained its strength almost entirely, while Panel E’s mean declined to roughly 40% of its ambient value, falling to approximately 25 N. Panel D occupied an intermediate position, retaining about 78%. The steep decline of Panel E under water immersion is consistent with moisture plasticization of the PP-based adhesive and the FRP-HPL interface, superimposed on the already-limited bonding area. These retention patterns are not discernible from ambient data alone and underscore the necessity of multi-condition aging for honeycomb-core qualification.

**Fig 5:**
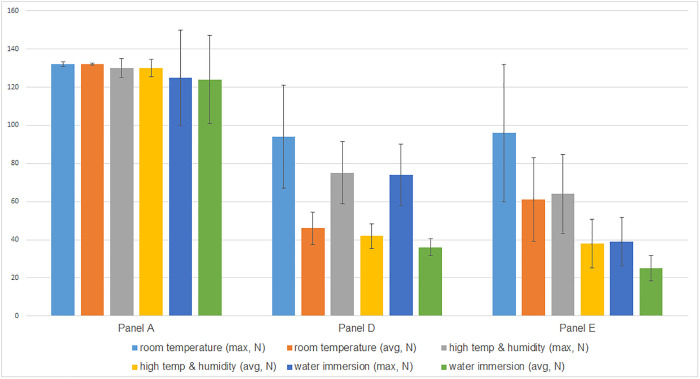
Edge‑band peel performance for Panels A, D and E under different environmental conditions. Average and maximum peel forces were measured using the internal peel‑test protocol on 150 × 100 mm specimens with a 150‑mm sealed edge. Bars represent the mean; error bars indicate standard deviation. Sample sizes: Panel A, n = 3 (1 batch) for all conditions; Panel D, n = 9 (1 batch) for all conditions; Panel E, ambient: n = 9 (2 batches), hygrothermal: n = 7 (2 batches), water immersion: n = 5 (1 batch). Full data are available in Supporting Information (Table B in S1 File).

Fig 5 presents maximum and average peel forces under ambient, hygrothermal (60 °C, 90% RH, 72 h), and water immersion (20 °C, 24 h) conditions; the complete per sample dataset is provided in the Supporting Information (Table B in S1 File).

An operational acceptance threshold of average ≥30 N or maximum ≥50 N was established from two sources: cumulative field data correlating peel strength with edge delamination in returned products, and benchmark measurements on commercially available competing tabletops (n = 3 per product, two batches). While the threshold is empirical and does not constitute a universal standard, it provides a practical, data-anchored gate for supplier qualification and internal design reviews.

The broader implication of these results is that edge peel variability is not merely a quality-control metric but a direct structural consequence of core architecture and adhesive selection. For foam-core panels, the bonding surface is inherently continuous, and variability is governed by process parameters such as adhesive application uniformity. For honeycomb-core panels, the bonding surface is inherently discontinuous, and variability is governed primarily by core geometry and surface energetics, factors that cannot be fully compensated by adhesive chemistry alone. The practical consequence is that industrialization of flat edge banding on honeycomb cores requires either (a) surface activation of the core edges to improve adhesive wetting, (b) local core densification or filler application in the edge zone, or (c) acceptance of higher process scrap as the cost of the desired aesthetic decisions that directly shape the cost-performance positioning of the final product.

### 3.3. Mechanical fastening strategies

Mechanical fastening was evaluated at two levels. The first was static vertical retention, represented by vertical pull‑out or screw‑holding capacity in Table 2. The second was product-level horizontal durability, represented by a stepped cyclic loading protocol in Table 3. These two tests are complementary rather than interchangeable. Static pull-out reflects the local anchoring capacity of the connection zone, whereas horizontal fatigue reflects whether the entire joint system can tolerate repeated shear transfer during folding without progressive loosening or failure.

**Table 2:** Static fastening performance of the five sandwich panel configurations.

Test Item	A PET Foam + Plywood	B Al‑Honeycomb + Bamboo	C Al‑Honeycomb + HPL	D Al‑Honeycomb + Al plate	E Plastic Honeycomb + FRP + HPL
Determination of resistance to axial withdrawal of screws (N)	474 ± 51	2455 ± 425			
Determination of pull-out resistance (N)	1721 ± 111	2663 ± 232	1776 ± 85	1721 ± 297 (insert) / 2386 ± 84 (rivet)	1621 ± 409 (insert) / 1602 ± 86 (rivet)

Values are mean ± SD. Screw withdrawal resistance was measured only for Panels A and B, which accept direct self‑tapping screws. Pull‑out resistance was measured for all panels; Panels D and E were tested with both bonded plastic inserts and blind rivets. Sample sizes: Panels A, B, C, and insert groups of D and E, one batch of three specimens each (n = 3). Panel D rivets, two batches of four specimens each (total n = 8). Panel E rivets, two batches of three specimens each (total n = 6).

**Table 3:** Horizontal fatigue performance of the five panel configurations under stepped cyclic loading.

Test step	A PET Foam + Plywood (n = 1)	B Al‑Honeycomb + Bamboo (n = 1)	C Al‑Honeycomb + HPL (n = 1)	D Al‑Honeycomb + Al plate (n = 3)	E Plastic Honeycomb + FRP + HPL (n = 5)
Camping condition: 100 N, 5000 cycles	1 / 1	1 / 1	1 / 1	3 / 3	5 / 5
Domestic condition: 150 N, 10000 cycles	1 / 1	1 / 1	1 / 1	3 / 3	5 / 5
Extended step: 200 N, 20,000 cycles	1 / 1	1 / 1	1 / 1	2 / 3^1^	3 / 5^2^
Contract step: 300 N, until failure	0 / 1^3^	1 / 1^4^	0 / 1^5^	0 / 3^6^	3 / 5^4^

Results are reported as pass/fail at each load step in the stepped staircase protocol. Each assembly was a complete folding table; Sample sizes varied by panel configuration: Panel A, n = 1; Panel B, n = 1; Panel C, n = 1; Panel D, n = 3; and Panel E, n = 5. Details of failures and test termination are given in the footnotes below.

^1^One specimen failed at 16779 cycles.

^2^Two specimens failed at 18799 and 19550 cycles, respectively.

^3^Screw pull‑out at 100 cycles.

^4^No failure; test stopped at 5000 cycles.

^5^Insert pull‑out at 520 cycles.

^6^The remaining two specimens failed at 1206 and 1349 cycles, respectively.

Among the directly screw-retained structures, Panel B showed the highest axial withdrawal resistance and pull-out resistance, consistent with its dense bamboo construction and favorable screw engagement. Panel A also provided stable static retention, but its foam-supported screw zone limited the amount of load-bearing material available around the fastener, so its withdrawal resistance remained much lower than Panel B. For the insert-based configurations, Panels C, D, and E showed that joint performance depended strongly on the connection concept rather than on the face sheet alone. Panel C achieved moderate pull-out resistance through its insert-supported architecture. Panel D showed a clear increase in pull-out resistance when the rivet was used instead of the insert, rising from 1721 ± 297 N to 2386 ± 84 N. In panel E the performance of the rivet configuration is comparable to that of the insert configuration. These results show that hardware redesign can improve static holding capacity, but static strength alone does not define the durability of the joint.

The staircase fatigue results in Table 3 make this limitation clear. Under the 100 N camping condition and the 150 N domestic condition, all panels passed the imposed cycles, showing that each concept is adequate for light and moderate service. Once the force increased to the 200 N extended step, differences began to emerge. Failures first appeared in some tests of Panels D and E, while Panels A, B, and C remained stable at that stage. At the final 300 N contract step, Panel A failed early by screw pull-out, and Panel C failed by insert pull-out. Panel B remained the most robust, with no failure within the imposed limit. The rivet-based configurations of Panels D and E showed mixed stability, indicating that they could improve static retention but still remained risk under repeated shear loading. The staircase protocol therefore revealed not only which joints were strong, but also which joint concepts lost stability first under progressively more strict service conditions.

A key result is that static pull-out strength did not correlate well with fatigue life. Panels D and E illustrate this clearly. Their rivet-based configurations produced relatively high pull-out values, yet fatigue failure still occurred under repeated horizontal loading. This means that the dominant failure mode in folding-table use is not simple fastener extraction, but cyclic damage accumulation in the full connection path. In practical terms, static pull-out mainly measures local clamping or bearing capacity, while fatigue performance depends on whether the fastener, connector, insert, and surrounding substrate can jointly sustain repeated shear transfer without progressive loosening. The failure sequence therefore reflects system-level durability rather than the strength of any single component.

The panel architecture also helps explain the observed differences. Panel B benefited from a mechanically dense fastening zone, which distributed the load more effectively around the screw engagement region. Panel A was limited by the lower density of the foam-supported screw zone. Panel C relied on an insert-based strategy that improved static retention but remained constrained by the bonded interface around the insert. The riveted assemblies of Panels D and E displayed high static pull‑out strengths, particularly Panel D at nearly 2400 N, yet failed earlier under cyclic loading. This occurs because rivets rely on clamping preload, which gradually relaxes through creep of the thermoplastic core or adhesive layers under repeated deformation. As preload diminishes, the joint loosens and the rivet head works against the face sheet, eventually pulling through. Static pull‑out, a monotonic test, cannot capture this creep‑driven sequence.

Post‑test examination of the fatigue specimens (S2 Fig in Supporting Information) revealed that failure consistently initiated at the central twist‑lock hub, not at the four corner inserts that are the subject of static pull‑out testing. Under horizontal cycling, the leg tubes transmit cyclic shear to this central hub, which experiences the highest cumulative displacement. Static pull‑out tests on corner inserts therefore probe a different load path and failure mode, explaining the poor correlation between the two metrics and identifying the central hub as the priority target for design improvement.

Overall, the fastening results show that outdoor folding tables must be qualified as complete joint systems rather than as isolated fasteners. Horizontal fatigue testing must be part of the qualification protocol, with particular attention to the central locking hub. For industrialization, the essential question is therefore not only whether a fastener can be installed easily, but whether the full load path can balance assembly efficiency, cyclic durability, and manufacturing cost. For mainstream products where assembly efficiency is critical, blind rivets offer an acceptable balance: despite earlier fatigue failure than solid‑insert systems, the majority of riveted assemblies survived 200 N/20 000 cycles, which exceeds expected field demands for portable tables. For premium applications requiring maximum fatigue life, solid‑insert or locally densified connection zones remain the more robust choice.

### 3.4. Material selection and cost-manufacturability trade-offs

Material selection governs not only structural performance but also environmental robustness, dimensional stability, mass, and production economics. The substrate-level results summarized in Table 4 show that no single architecture dominates all criteria simultaneously. Instead, each panel occupies a distinct design position defined by the interaction between face-sheet toughness, core continuity, interfacial stability, and manufacturing complexity.

**Table 4:** Mechanical and environmental performance of core and substrate systems.

Test Item	A PET Foam + Plywood	B Al‑Honeycomb + Bamboo	C Al‑Honeycomb + HPL	D Al‑Honeycomb + Al plate	E Plastic Honeycomb + FRP + HPL
Determination of impact resistance with small ball	No abnormality	No abnormality	Damage	Denting only	No abnormality
Modulus of rupture (MPa)	23.3 ± 2.9	27.7 ± 3.5	32.2 ± 1.7	25.9 ± 1.0	23.9 ± 2.2
Modulus of rupture, high temp & humidity (MPa)	16.6 ± 1.4	20 ± 1.0	31 ± 0.8	25.3 ± 0.9	19.2 ± 2.6
Water-immersion test for bond durability					
condition c	Delamination	Delamination	No abnormality	No abnormality	No abnormality
condition b	Not continued	Not continued	No abnormality	No abnormality	No abnormality
condition a	Not continued	Not continued	No abnormality	No abnormality	No abnormality
Heat-induced deformation	4 mm sag; pass	Pass	Pass	Pass	2 mm sag; pass

Values are mean ± SD where applicable. Sample sizes: Impact resistance: Panels A, B, C: n = 3 (1 batch each); Panel D, E: n = 3 (2 batches each). MOR, room temperature: Panels A, B, C: n = 3 (1 batch each); Panel D: n = 6 (2 batches); Panel E: n = 5 (3 batches). MOR, hygrothermal aged: Panels A, B, C: n = 3 (1 batch each); Panel D: n = 6 (2 batches); Panel E: n = 5 (2 batches). Water immersion durability, n = 3 each: condition c: Panel A, 3 batches; Panels B, C, D: 1 batch each; Panel E: 2 batches; conditions b and a: Panels C, D: 1 batch each; Panel E: 2 batches. Heat-induced deformation, n = 1 each: Panel A: 2 batches; Panels B, C, D: 1 batch each; Panel E: 4 batches. “Not continued” indicates that the panel had already failed at a milder condition.

Impact resistance was governed by the face-sheet system rather than the core. Panels with thick or fiber-reinforced skins, such as Panel A (plywood), B (bamboo veneer), and E (FRP), showed no visible damage or only minor cosmetic marking. Panel C, with its thin, brittle HPL face, sustained visible damage under the same impact energy, while Panel D’s aluminum face dented. This pattern is consistent with the mechanics of low-velocity impact on sandwich structures: the face sheet absorbs the initial contact energy, and its thickness and intrinsic ductility determine whether the underlying core is shielded. For outdoor tabletops, where incidental impacts from dropped objects are routine, skin toughness represents a practical gating criterion.

Modulus of rupture (MOR) is an important proxy for perceived tabletop rigidity. All five panels exceeded the empirically derived threshold of approximately 20 MPa for adequate stiffness at the tested thicknesses, with room-temperature MOR ranging from 23.3 ± 2.9 MPa (Panel A) to 32.2 ± 1.7 MPa (Panel C). The relatively narrow spread indicates that within the thickness range typical of portable tables, the sandwich configuration itself, rather than the specific core material, is the primary determinant of static rigidity. After hygrothermal conditioning (60 °C, 90% RH, 72 h), differentiation sharpened. Panels C and D, with aluminum honeycomb cores and low-hygroscopicity face sheets, retained over 95% of their room-temperature MOR. Panel E retained 80%, with the reduction concentrated at the adhesive interfaces rather than in the hydrophobic PP core itself. Panel A and B retained only about 70%, reflecting the moisture sensitivity of the wood-based faces and the core-wood bondline. This pattern indicates that hygrothermal durability is governed by moisture uptake, interfacial relaxation, and thermal expansion mismatch. Wood-based or moisture-active systems lose stiffness more readily because dimensional change in the substrate transfers directly into the adhesive interfaces. In contrast, metal-faced sandwich structures preserve load transfer more effectively because their face sheets are less sensitive to moisture and thermal softening. These differences are not observable from ambient data alone and underscore the necessity of environmental conditioning as a standard element of industrialization qualification.

Water immersion confirms the same structural logic from a different angle. Panels A and B delaminated under the severe immersion sequence, while Panels C, D, and E remained intact. This is not simply a matter of whether a panel contains wood. It reflects the combined influence of substrate hygroscopicity, bondline continuity, and the ability of the structure to resist moisture-assisted interfacial weakening. In Panels A and B, moisture-sensitive constituents and adhesive interfaces are more exposed to swelling, softening, and progressive bond degradation once water penetration occurs. Panels C and D, which rely on more dimensionally stable face systems, are less prone to this failure pathway. Panel E also remained stable in the immersion test, but its lower retained MOR and greater sensitivity in other tests show that passing a short-term immersion screen does not automatically guarantee balanced long-term mechanical robustness. Water resistance and bending retention are therefore related but not identical quality dimensions.

Heat-induced deformation revealed a clear distinction among the five architectures. Panels B, C, and D exhibited negligible deformation and passed their respective tests without measurable sag, while Panels A and E showed quantifiable deflection. Panel A developed 4 mm of sag across two tests, and Panel E showed 2 mm across four independent tests. This indicates that the latter structures retained substantially better flatness under one-sided radiant heating. The pass criterion of maximum deflection ≤ 0.5% of panel length was selected because warpage at or above this level becomes visually noticeable and can affect perceived tabletop quality even before structural failure occurs. The measurable sag in Panel A suggests greater sensitivity to thermal expansion mismatch and skin compliance, whereas the lower deformation of Panels B, C, and D reflects the superior thermal stability of aluminum honeycomb, which combines low thermal expansion with high specific stiffness. For industrialization, these results indicate that foam-core panels require either a thicker core section or a higher-density foam grade if flatness under solar exposure is critical, whereas both aluminum and PP honeycomb architectures provide adequate thermal shape stability for outdoor tabletop service.

From an industrialization perspective, not all material parameters carry equal leverage. The data across Sections 3.1–3.4 reveal a clear hierarchy. Core type is the dominant decision: it simultaneously governs panel weight, static rigidity, edge-bonding difficulty (continuous foam vs. discontinuous honeycomb), moisture durability, and material cost-typically accounting for an estimated 40–50% of the unit bill of materials in the configurations studied. Face-sheet material and thickness constitute the second tier, primarily controlling impact resistance, surface thermal performance, and fastening substrate quality. Surface finishing chemistry, while critical for weatherability and consumer perception, has a comparatively minor influence on structural performance. This hierarchy implies that industrialization efforts should prioritize core selection and core-face bonding optimization, as downstream performance in edge sealing and fastening are largely consequences of these upstream choices.

The product-level attributes in Table 5 translate these technical trade-offs into manufacturability and positioning terms. Panel E achieves the lowest weight (3669 g) and lowest relative cost (index 1.00) among the five designs, reflecting the inherently lower material cost and shorter cycle times of PP honeycomb processing. Panel B, at over 2.5 times the cost of Panel E, delivers premium aesthetics and the highest static fastening strength but imposes a significant weight penalty. Panel D occupies an intermediate position: its cost index of 1.75 reflects the combined expense of aluminum honeycomb core and aluminum face sheets, but its low process variability and high environmental stability reduce the risk of post-production quality costs.

**Table 5:** Product‑level attributes, including dimensions, weight, and relative cost.

Test Item	A PET Foam + Plywood	B Al‑Honeycomb + Bamboo	C Al‑Honeycomb + HPL	D Al‑Honeycomb + Al plate	E Plastic Honeycomb + FRP + HPL
Dimensions (mm)	1200*800*18.5	1150*700*18	1150*700*16	1150*700*18	1152*702*16.4
Weight (g)	6400	6760	3740	4200	3669
Relative Cost index* (RMB/pcs)	1.53	2.50	1.14	1.75	1.00

* Relative cost index was normalized to the lowest-cost prototype in this study (Panel E = 1.00). The index reflects the combined material and processing cost tendency of the final prototypes and is intended for internal engineering comparison rather than market pricing.

The fundamental industrialization conclusion is that no single panel architecture simultaneously maximizes durability, manufacturability, portability, and cost control. Instead, the five configurations define a performance-cost frontier from which product-specific choices can be made. For mass-market portable tables where price sensitivity dominates, Panel E’s combination of adequate mechanical performance, superior moisture resistance, and lowest cost makes it the rational baseline, with the caution that edge-bonding process control must be tightened to manage its higher peel variability. For premium segments where edge aesthetics and tactile quality are differentiating features, Panel D’s low process variability and Panel B’s natural material appeal justify their cost premiums, though Panel B’s moisture sensitivity restricts it to temperate climates or covered outdoor use. These trade-offs are not theoretical; they directly inform the tiered product strategy and supplier qualification protocols that constitute the industrialization roadmap for sandwich composite outdoor tabletops.

### 3.5. Summary

Taken together, the results demonstrate that industrializing sandwich composite panels for portable outdoor tabletops requires a balanced integration of surface durability, edge protection, fastening reliability, and manufacturable material combinations. Across all four challenge domains, the testing matrix clarified which performance gaps can be resolved through engineering controls, such as adhesive optimization, fastening reinforcements, and edge‑banding process refinement, and which limitations are intrinsic to specific material systems. Although no single panel configuration achieves absolute superiority, the study provides a practical decision framework that connects mechanical performance, environmental durability, weight, aesthetics, and cost to different market tiers. These insights establish a technical foundation for transitioning sandwich structures from high‑end sectors such as aerospace and transportation into mainstream outdoor consumer products.

## 4. Conclusion

This study set out to identify and validate the key technologies required to industrialize sandwich composite panels for portable outdoor table applications. By integrating customer‑driven requirements, applicable standards, and internally developed evaluation methods, we established a comprehensive testing matrix that covered surface durability, edge sealing, fastening strategies, and material-process trade‑offs. Five representative panel constructions were fabricated under controlled prototype conditions and evaluated through both material‑level and product‑level tests, providing a multi‑dimensional assessment of performance under realistic outdoor stressors.

The findings confirm that sandwich composites can meet the structural and environmental demands of portable outdoor tabletops when several critical engineering factors are addressed. These include fastening fatigue resistance, edge‑banding integrity, and adhesive‑interface stability under temperature and humidity gradients. The results also show that different material systems are suited to different product tiers. Lightweight synthetic skins are appropriate for portability‑focused mass‑market products, whereas wood‑based skins are better aligned with premium, aesthetics‑driven designs.

Beyond evaluating individual panel configurations, this work contributes a generalizable framework for industrializing sandwich structures in consumer outdoor products. The framework consists of three elements: defining tier‑appropriate performance thresholds, validating performance through multi‑mode accelerated tests, and managing manufacturability constraints through process‑specific controls. Future work should include long‑term outdoor exposure trials, multi‑batch statistical validation, and studies on process automation. These steps are essential for scaling sandwich composite technology from prototype development to stable, high‑volume production.

## Supporting information

S1 Fig Failure modes observed in pull-out resistance testing. Representative images showing failure modes during pull-out resistance testing: (A) pull‑out of a self‑tapping screw; (B) pull‑out of a rivet; (C and D) failures involving a plastic embedded insert combined with a self‑tapping screw, showing screw pull‑out and local substrate damage around the fastening zone.
(TIF)

S2 Fig Failure progression during horizontal fatigue staircase testing. Images document progressive damage and final failure states for folding table assemblies under horizontal fatigue step loading: (A) specimen on the test fixture at the moment of failure; (B) magnified detail of the detached area; (C) post‑test lateral view of the table assembly; (D) post‑test underside view highlighting damage to the fastening region.
(TIF)

S1 File Supporting Information. Contains captions / descriptions of S1 and S2 Fig, Table A (non-proprietary material specifications), and Table B (edge peel strength data with statistics).
(DOCX)

## References

[1] Jang WC, Roh HD. Sandwich composites manufacturing: A review of materials, methods, applications and challenges. Int J Precis Eng Manuf. 2025;26:2093–109. doi: 10.1007/s12541-025-01285-8

[2] Hoff NJ, Mautner SE. The Buckling of Sandwich-Type Panels. J Aeronaut Sci. 1945;12(3):285–97. doi: 10.2514/8.11246

[3] Sahu SK, Sreekanth PSR, Reddy SVK. A Brief Review on Advanced Sandwich Structures with Customized Design Core and Composite Face Sheet. Polymers (Basel). 2022;14(20):4267. doi: 10.3390/polym14204267 36297845 PMC9608463

[4] Gibson RF. Principles of composite material mechanics. CRC Press; 2010.

[5] Castanie B, Bouvet C, Ginot M. Review of composite sandwich structure in aeronautic applications. Composites Part C: Open Access. 2020;1.

[6] Vosteen LF, Hadcock RN. Composite chronicles: a study of the lessons learned in the development, production, and service of composite structures (No. NASA-CR-4620). NASA; 1994.

[7] Palomba G, Epasto G, Crupi V. Lightweight sandwich structures for marine applications: a review. Mech Adv Mater Struct. 2022;29(26):4839–64. doi: 10.1080/15376494.2021.1941448

[8] Han Z, Jang J, Souppez J-BRG, Maydison, Oh D. Environmental implications of a sandwich structure of a glass fiber-reinforced polymer ship. Ocean Eng. 2024;298:117122. doi: 10.1016/j.oceaneng.2024.117122

[9] Abhinav SN, Budharaju MV. A review paper on origin of honeycomb structure and its sailing properties. Int J Eng Res Technol. 2020;9(8):861–6.

[10] Dequine DL, Rush C. Continuous fiber composite part cost vs production volume by manufacturing process and material. Lockheed Martin; 2014.

[11] Akiyama K. Development of PCM (Prepreg Compression Molding) Technology. In: Automotive Lightweight Procurement Symposium. Dusseldorf; 2014.

[12] Williams C, Summerscales J, Grove S. Resin Infusion under Flexible Tooling (RIFT): a review. Compos A: Appl Sci Manuf. 1996;27(7):517–24. doi: 10.1016/1359-835x(96)00008-5

[13] Gupta MK, Singhal V. Review on materials for making lightweight vehicles. Mater Today Proc. 2022;56:868–72. doi: 10.1016/j.matpr.2022.02.517

[14] Wan Y, Takahashi J. Development of Carbon Fiber-Reinforced Thermoplastics for Mass-Produced Automotive Applications in Japan. J Compos Sci. 2021;5(3):86. doi: 10.3390/jcs5030086

[15] Leong M, Overgaard LCT, Thomsen OT, Lund E, Daniel IM. Investigation of failure mechanisms in GFRP sandwich structures with face sheet wrinkle defects used for wind turbine blades. Composite Structures. 2012;94(2):768–78. doi: 10.1016/j.compstruct.2011.09.012

[16] Fortune Business Insights. Outdoor Furniture Market Size, Share & Industry Analysis, By Product Type, By Material Type, By End-User, and Regional Forecasts, 2025-2032. [accessed 2026 February 09]. Available from: https://www.fortunebusinessinsights.com/outdoor-furniture-market-106406

[17] Techawinyutham L, Ayyappan V, Kumar M, Raghunathan V, Rangappa SM, Siengchin S. Sustainable epoxy composites from hemp/pineapple/glass fibers for lightweight automobile panels. Int J Biol Macromol. 2026;337(Pt 1):149392. doi: 10.1016/j.ijbiomac.2025.149392 41344458

[18] Patel D, Bhat S. Portable Outdoor Furniture Market Global Industry Analysis, Size, Share, Growth, Trends and Forecast 2025-2033. Dataintelo. [accessed 2026 February 24]. Available from: https://dataintelo.com/report/portable-outdoor-furniture-market#

[19] Hörmann S. Sieben Campingtische im Vergleich: Ist ein Falt- oder Rolltisch besser geeignet fürs Camping? Promobil. [accessed 2020 August 8]. Available from: https://www.promobil.de/zubehoer/campingtisch-vergleichstest-roll-falttisch-zubehoer/

[20] Yorseng K, Srisuk R, Ayyappan V, Thirugnanasamabandam A, Rangappa SM, Siengchin S. Lightweight innegra-hemp/epoxy hybrid composites: Effects of weathering on mechanical, thermal, and viscoelastic properties. Int J Precis Eng Manuf. 2026;27:1025–41. doi: 10.1007/s12541-025-01377-5

[21] Studziński R. Experimental investigation of the use of blind rivets in sandwich panels. J Sandwich Struct Mater. 2020;23(8):3669–84. doi: 10.1177/1099636220936146

[22] Jiang Q, Chen G, Kumar A, Mills A, Jani K, Rajamohan V, et al. Sustainable Sandwich Composites Manufactured from Recycled Carbon Fibers, Flax Fibers/PP Skins, and Recycled PET Core. J Compos Sci. 2020;5(1):2. doi: 10.3390/jcs5010002

[23] Mandegarian S, Hojjati M, Moghaddar H. Thermoplastic composite sandwich panels with recycled PET foam core: A manufacturing process assessment. J Thermoplast Compos Mater. 2024;38(5):1886–913. doi: 10.1177/08927057241291021

[24] Friedrich K, Almajid AA. Manufacturing Aspects of Advanced Polymer Composites for Automotive Applications. Appl Compos Mater. 2012;20(2):107–28. doi: 10.1007/s10443-012-9258-7

[25] Ayyappan V, Arora G, Kumar M, Raghunathan V, Rangappa SM, Siengchin S. Appl Sci Eng Prog. 2025;18(4):7883. doi: 10.14416/j.asep.2025.07.001

[26] Wei P, Chen J, Zhang Y, Pu L. Wood-based Sandwich Panels: A Review. Wood Res. 2021;66(5):875–90. doi: 10.37763/wr.1336-4561/66.5.875890

[27] Vladimirova E, Gong M. Advancements and Applications of Wood-Based Sandwich Panels in Modern Construction. Buildings. 2024;14(8):2359. doi: 10.3390/buildings14082359

