# Supplementary figures and images for: Industrialization of sandwich composite panels for portable outdoor tabletops
Source: PLoS One. 2026 Jul 9;21(7):e0353571. doi: 10.1371/journal.pone.0353571 (PMC13349161; doi:10.1371/journal.pone.0353571)

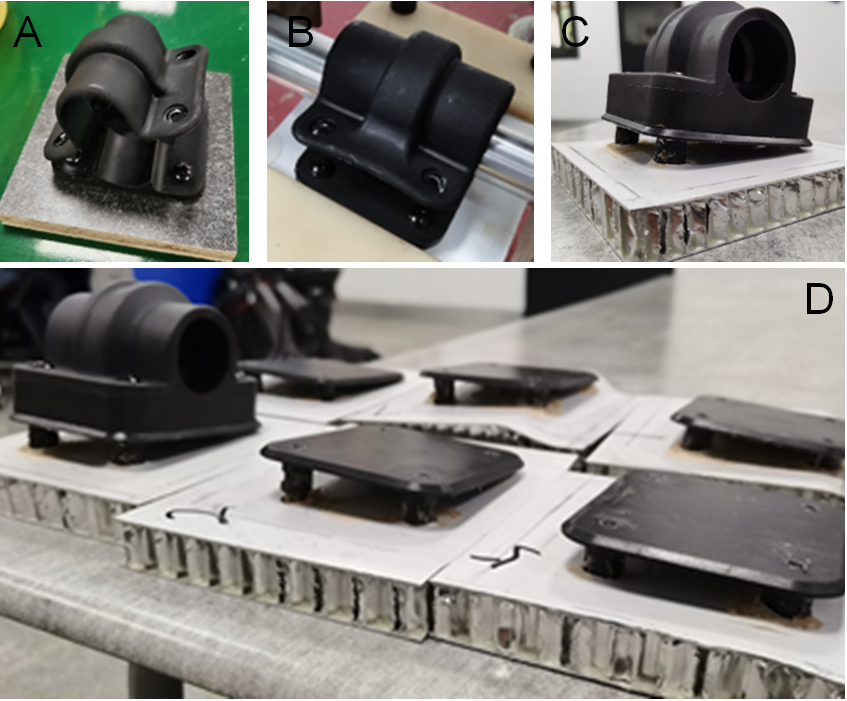

Supplement: S1 Fig — Representative images showing failure modes during pull-out resistance testing: (A) pull‑out of a self‑tapping screw; (B) pull‑out of a rivet; (C and D) failures involving a plastic embedded insert combined with a self‑tapping screw, showing screw pull‑out and local substrate damage around the fastening zone. (TIF) [file pone.0353571.s001.tif]

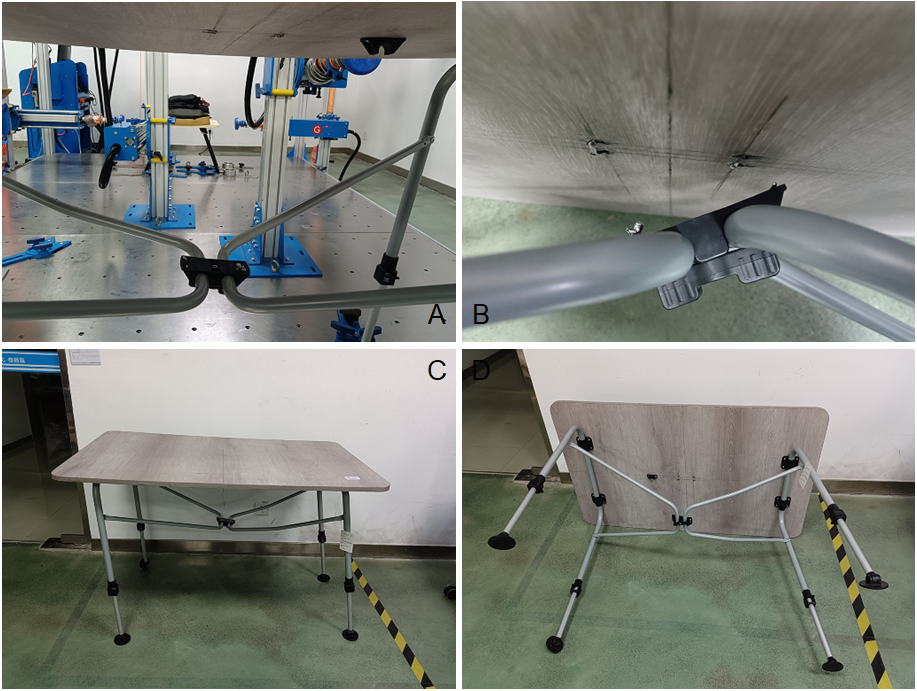

Supplement: S2 Fig — Images document progressive damage and final failure states for folding table assemblies under horizontal fatigue step loading: (A) specimen on the test fixture at the moment of failure; (B) magnified detail of the detached area; (C) post‑test lateral view of the table assembly; (D) post‑test underside view highlighting damage to the fastening region. (TIF) [file pone.0353571.s002.tif]
