# Supplementary material for: Industrialization of sandwich composite panels for portable outdoor tabletops
Source: PLoS One. 2026 Jul 9;21(7):e0353571. doi: 10.1371/journal.pone.0353571 (PMC13349161; doi:10.1371/journal.pone.0353571)
Supplement: S1 File — Contains captions / descriptions of S1 and S2 Fig, Table A (non-proprietary material specifications), and Table B (edge peel strength data with statistics). (DOCX) [file pone.0353571.s003.docx]

# Supporting Information

S1_Fig.tif

S1 Fig. Failure modes observed in pull-out resistance testing. Representative images showing failure modes during pull-out resistance testing: (A) pull‑out of a self‑tapping screw; (B) pull‑out of a rivet; (C and D) failures involving a plastic embedded insert combined with a self‑tapping screw, showing screw pull‑out and local substrate damage around the fastening zone.

S2_Fig.tif

S2 Fig. Failure progression during horizontal fatigue staircase testing. Images document progressive damage and final failure states for folding table assemblies under horizontal fatigue step loading: (A) specimen on the test fixture at the moment of failure; (B) magnified detail of the detached area; (C) post‑test lateral view of the table assembly; (D) post‑test underside view highlighting damage to the fastening region.

Table A. Non‑proprietary material specifications for the five prototype sandwich‑panel configurations. Reported values include core density ranges, face sheet thickness ranges, and nominal total panel thickness. These ranges reflect commercially available material classes and batch to batch variation observed during prototyping. Exact supplier specific formulations, adhesive chemistries, and processing parameters remain confidential under industrial agreements. The reported ranges are sufficient to reproduce the architectural concepts and to interpret the structure–property relationships discussed in the manuscript.

| ID | Core Family | Core density range (kg/m³) | Face Family | Face‑sheet thickness range (mm) | Total thickness (mm) | Edge Treatment | Finish | Fastening Approach |
| --- | --- | --- | --- | --- | --- | --- | --- | --- |
| A | Closed‑cell PET foam | 80–120 | Exterior plywood | 4–6 | 18.5 | Adhesive seal | Melamine‑impregnated paper | Direct self‑tapping screws |
| B | Aluminum honeycomb | 41-83 | Bamboo veneer | 3–5 | 18 | Bamboo veneer | Clear lacquer | Embedded wood/block reinforcement (screw) |
| C | Aluminum honeycomb | 41-83 | HPL laminate | 1–2 | 16 | Wrapped metal profile | Melamine‑impregnated paper | Plastic embedded inserts → screws |
| D | Aluminum honeycomb | 70-111 | Aluminum sheet | 0.8–1.2 | 18 | Adhesive seal | UV‑cured print on metal | Blind rivets / plastic embedded inserts |
| E | Polymeric honeycomb (PP) | 90–140 | FRP + HPL overlay | 0.9–1.4 | 16.4 | Adhesive seal or wrapped metal profile | Melamine‑impregnated paper | Blind rivets / plastic embedded inserts |

Table B. Edge sealing performance under room temperature, hygrothermal, and water immersion conditions. Values are reported as mean ± standard deviation. CV (coefficient of variation) quantifies repeatability, and the retained ratio represents the percentage of room temperature peel strength preserved after conditioning. Standard deviations are reported with one additional decimal place relative to the mean to preserve meaningful precision, as several SD values are small in magnitude. This level of numerical detail supports statistical transparency without disclosing proprietary adhesive formulations or processing parameters.

| Test Item | A PET Foam + Plywood | n (batch) | CV (%) | ratained (%) | D Al‑Honeycomb + Al plate | n (batch) | CV (%) | ratained (%) | E Plastic Honeycomb + FRP + HPL | n (batch) | CV (%) | ratained (%) |
| --- | --- | --- | --- | --- | --- | --- | --- | --- | --- | --- | --- | --- |
| room temperature (max, N) | 132 ± 1.2 | 3 (1) | 0.9% | - | 94 ± 27.0 | 9 (1) | 28.7% | - | 96 ± 36.0 | 9 (2) | 37.5% | - |
| room temperature (avg, N) | 132 ± 0.6 | 3 (1) | 0.5% | - | 46 ± 8.6 | 9 (1) | 18.7% | - | 61 ± 22.0 | 9 (2) | 36.1% | - |
| high temp & humidity (max, N) | 130 ± 5.0 | 3 (1) | 3.8% | 98% | 75 ± 16.3 | 9 (1) | 21.7% | 80% | 64 ± 20.6 | 7 (2) | 32.2% | 67% |
| high temp & humidity (avg, N) | 130 ± 4.6 | 3 (1) | 3.5% | 98% | 42 ± 6.5 | 9 (1) | 15.5% | 91% | 38 ± 12.8 | 7 (2) | 33.7% | 62% |
| water immersion (max, N) | 125 ± 25.0 | 3 (1) | 20.0% | 95% | 74 ± 16.0 | 9 (1) | 21.6% | 79% | 39 ± 12.7 | 5 (1) | 32.6% | 41% |
| water immersion (avg, N) | 124 ± 23.0 | 3 (1) | 18.5% | 94% | 36 ± 4.4 | 9 (1) | 12.2% | 78% | 25 ± 6.6 | 5 (1) | 26.4% | 41% |
